# MRI-based intratumoral and peritumoral radiomics for assessing deep myometrial invasion in patients with early-stage endometrioid adenocarcinoma

**DOI:** 10.3389/fonc.2024.1474427

**Published:** 2025-01-15

**Authors:** Jing Yang, Yang Liu, Xiaolong Liu, Yaoxin Wang, Xianhong Wang, Conghui Ai, Qiu Bi, Ying Zhao

**Affiliations:** ^1^ Department of MRI, The First People’s Hospital of Yunnan Province, The Affiliated Hospital of Kunming University of Science and Technology, Kunming, Yunnan, China; ^2^ Department of Radiology, The First Affiliated Hospital of Chongqing Medical University, Chongqing, China; ^3^ Department of Medical Imaging, The People’s Hospital of Puer, The Affiliated Hospital of Kunming University of Science and Technology, Puer, Yunnan, China; ^4^ Department of Radiology, Shenzhen Traditional Chinese Medicine Hospital, The Fourth Clinical Medical College of Guangzhou University of Chinese Medicine, Shenzhen, China; ^5^ The Affiliated Hospital of Kunming University of Science and Technology, School of Clinical Medicine, Kunming, Yunnan, China; ^6^ Department of Radiology, Yunnan Cancer Hospital, The Third Affiliated Hospital of Kunming Medical University, Peking University Cancer Hospital Yunnan, Kunming, Yunnan, China

**Keywords:** endometrial carcinoma, deep myometrial invasion, MRI, radiomics, peritumoral

## Abstract

**Purpose:**

To evaluate the effectiveness of magnetic resonance imaging (MRI)-based intratumoral and peritumoral radiomics models for predicting deep myometrial invasion (DMI) of early-stage endometrioid adenocarcinoma (EAC).

**Methods:**

The data of 459 EAC patients from three centers were retrospectively collected. Radiomics features were extracted separately from the intratumoral and peritumoral regions expanded by 0 mm, 5 mm, and 10 mm on unimodal and multimodal MRI. Then, various radiomics models were developed and validated, and the optimal model was confirmed. Integrated models were constructed by ensemble and stacking algorithms based on the above radiomics models. The models’ performance was evaluated using the area under the curve (AUC).

**Results:**

The multimodal MRI-based radiomics model, which included both intratumoral and peritumoral regions expanded by 5 mm, was the optimal radiomics model, with an AUC of 0.74 in the validation group. When the same integrated algorithm was utilized, the integrated models with 5-mm expansion presented higher AUCs than those with 0-mm and 10-mm expansion in the validation group. The performance of the stacking model and ensemble model with 5-mm expansion was similar, and their AUCs were 0.74 and 0.75, respectively.

**Conclusion:**

The multimodal radiomics model from the intratumoral and peritumoral regions expanded by 5 mm has the potential to improve the performance for detecting DMI of early-stage EAC. The integrated models are of little value in increasing the prediction.

## 1 Introduction

Endometrial cancer (EC) is the sixth most commonly diagnosed cancer in women (1), and with the increasing obesity rate, the incidence and disease-associated mortality are also increasing (2). Endometrioid adenocarcinoma (EAC) accounts for 75% to 80% of EC cases (3). According to the staging system of the International Federation of Gynecology and Obstetrics (FIGO), 75% of cases are in stage I at the time of detection, and EC can be further classified into stages IA and IB depending on the depth of myometrial invasion (4). Deep myometrial invasion (DMI), defined as the depth of infiltration ≥50% of the thickness of the myometrium, is considered to be one of the most important factors affecting the prognosis of EC, as tumors with DMI have a greater probability of paracervical invasion and pelvic lymph node metastasis (5, 6). Hence, DMI can be used as a marker to identify possible lymph node metastasis and the risk of lymphovascular space invasion (5, 7). The primary treatment of stage I EC is total hysterectomy with bilateral salpingectomy (8). The depth of myometrial invasion is a key factor in determining the operation mode of EAC (5). DMI status will determine whether ovaries can be preserved in young patients and whether lymph nodes need to be removed (8). Therefore, it is of great significance for preoperative detection to determine the depth of myometrial infiltration, which will help gynecologists develop an appropriate treatment plan and avoid overtreatment of EAC patients. Preoperative detection of the depth of myometrial infiltration is of great significance.

Magnetic resonance imaging (MRI) has significant advantages in assessing the depth of myometrial invasion of EC (9), which has become the primary method for preoperative assessment of the depth of myometrial infiltration of EC (10). In conventional MRI, T2-weighted imaging (T2WI) has important value in assessing the depth of myometrial invasion in EC patients (11, 12). Dynamic contrast-enhanced MRI (DCE-MRI) or diffusion-weighted imaging (DWI) and apparent diffusion coefficient (ADC) combined with T2WI have been demonstrated to detect DMI of EC (13, 14). However, the performance of MRI for assessing DMI of EC depends on the professional knowledge and subjective experience of radiologists (15). Moreover, there are huge differences in the agreement and diagnostic accuracy of different radiologists when assessing DMI (16). In addition, morphological evaluation is challenging to accurately detect DMI in the absence of a definition of the borderline and poor impact of tumors on the myometrium (17).

Radiomics is a precise and non-invasive approach that converts MR images to mineable data into high-dimensional data and subsequently analyzes the data to offer abundant information on EC (18), including intertumoral and peritumoral information, which could be a supplement to conventional images or clinical data (19). At present, there are some articles on the assessment of DMI based on intratumoral radiomics (20, 21), but few articles use radiomics combining intratumoral features with peritumoral features to evaluate DMI of EC (22).

The purpose of this study was to establish the various intratumoral and peritumoral radiomics models on unimodal and multimodal MRI for predicting DMI in early-stage EAC, and unimodal and multimodal radiomics models were fused using different integration algorithms.

## 2 Materials and methods

### 2.1 Patient selection

The ethical approval of three clinical centers approved this retrospective study. The informed consent was waived. This study collected preoperative MR images and clinical data of patients with EAC from January 2017 to June 2023. The inclusion criteria were as follows: 1) stage I EAC patients confirmed by surgery and pathology with complete clinical data, 2) patients with satisfactory imaging quality, and 3) MR images including T2WI, DWI, and late contrast-enhanced T1-weighted imaging (LCE-T1WI) within 2 weeks before surgery. The exclusion criteria were as follows: 1) patients with a history of other malignant tumors, 2) the maximum diameter of the tumor was less than 10 mm, and 3) underwent surgery, chemoradiotherapy, or other treatment before MRI examination.

### 2.2 Clinical parameters

The clinical characteristics including age, menopausal status, metabolic syndrome (including hypertension, diabetes, or hyperlipidemia), body mass index (BMI), tumor grade, preoperative cancer antigen 125 (CA125), and preoperative cancer antigen 199 (CA199) were obtained from the medical record system. A total of 459 patients (aged 53.21 to 9.19 years) were included in the study. Of them, 281 patients from center A were assigned to the training group (222 patients with stage IA and 59 patients with stage IB); 71 patients from center B were assigned to validation group A (41 patients with stage IA and 30 patients with stage IB); 107 patients from center C were assigned to validation group B (78 patients with stage IA and 29 patients with stage IB).

### 2.3 MRI acquisition

All MRI examinations were performed using 1.5/3.0-T scanners with 8-channel sensitivity encoding phased-array abdominal coils. In this study, oblique axial T2WI, DWI (b-value = 1,000 s/mm^2^), ADC map, and LCE-T1WI were selected. LCE-T1WI images were obtained approximately 3 minutes after intravenous gadolinium administration (0.1 mL/kg, Magnevist; Bayer Pharmaceutical Company, Schönefeld, Germany) at a rate of 2 mL/s. **Table 1** shows the parameters of the selected sequences of each MR scanner.

**Table 1:** The parameter details of primary sequences.

		Sequence	Repetition time (ms)	Echo time (ms)	Field of view (mm^2^)	Acquisition matrix (ms)	Slice thickness (mm)	Slice gap (mm)
Training group	Siemens Aera 1.5 T	T2WI	3,900	90	320 × 320	512 × 512	3	1.5
		DWI (b = 0 and 1,000 s/mm^2^)	5,600	90	200 × 200	256 × 256	4	1
		LCE-T1WI	3.41	1.3	240 × 240	320 × 320	2	1.5
	Siemens Prisma 3.0 T	T2WI	3,200	90	200 × 200	320 × 320	3	3.6
		DWI (b = 0 and 1,000 s/mm^2^)	6,300	75	250 × 134	72 × 134	3	3.6
		LCE-T1WI	2.9	1.19	220 × 200	288 × 262	3	0
	GE Signa HDXt 3.0T	T2WI	3,500	104	200 × 200	240 × 240	3	1.5
		DWI (b = 0 and 1,000 s/mm^2^)	4,250	70	200 × 200	240 × 240	3	1
		LCE-T1WI	3.26	1.6	240 × 240	350 × 350	3	1.5
Validation group A	GE Signa HDXt 3.0T	T2WI	3,500	104	200 × 200	240 × 240	3	1.5
		DWI (b = 0 and 1,000 s/mm^2^)	4,250	70	200 × 200	240 × 240	3	1
		LCE-T1WI	3.26	1.6	240 × 240	350 × 350	3	1.5
Validation group B	Philips Ingenia 3.0T	T2WI	1,835	100	200 × 200	332 × 284	3	0.3
		DWI (b = 0 and 1,000 s/mm^2^)	5,271	55	200 × 250	80 × 98	3	0.3
		LCE-T1WI	3.7	1.32	400 × 353	288 × 253	5	−2.5
	GE Pioneer 3.0T	T2WI	4,904	85	200 × 200	320 × 256	3	0.3
		DWI (b = 0 and 1,000 s/mm^2^)	3,675	Minimum	180 × 144	110 × 72	3	0.3
		LCE-T1WI	3.5	1.7	400 × 360	340 × 256	5	−2.5

T2WI, T2-weighted imaging; DWI, diffusion-weighted imaging; LCE-T1WI, late contrast-enhanced T1-weighted imaging.

### 2.4 Image segmentation

Image segmentation was carried out using the 3D Slicer 4.11.0 (https://www.slicer.org/) software. Rigid registration was used to match the multisequence pictures of the axial oblique T2WI, DWI, ADC, and LCE-T1WI to ensure spatial coherence within a shared reference space. The poorly matched sequences were delineated separately in each sequence. The tumor boundary was manually delineated on the T2WI sequence layer by layer with reference to other sequences. The normal myometrium adjacent to the tumor was avoided, but the region of interest (ROI) comprised bleeding, necrotic, or cystic areas. The ROIs included intratumoral regions (expanded by 0 mm) and intratumoral combined with peritumoral regions expanded by 5 mm and 10 mm (22–24).

The segmentation of the tumors was performed by two radiologists (radiologist A and radiologist B, with 5 years and 9 years of experience in pelvic MRI, respectively) who were blinded to the histopathological results. Radiologist A redrew each patient’s ROI after 2 months. To assess consistency among and within observers, the intraclass correlation coefficient (ICC) of each characteristic was determined. Features with an inter-observer consistency of less than 0.75 were disregarded.

### 2.5 Feature extraction and selection

Image preprocessing and feature extraction were performed using Pyradiomics (https://pypi.org/project/pyradiomics/). Radiomics features from the ROIs on T2WI, DWI, ADC, LCE-T1WI, and multimodal MRI (combining the four sequences) were extracted. To create isotropic voxels, the MR images and ROIs were resampled to 3 × 3 × 3 mm, and cubic spline interpolation was carried out. By subtracting the average values, dividing by the standard deviation, multiplying the values by 100, and adding a 300-voxel array shift, the intensity values were normalized. The distribution of gray-level intensity in the photos falls between 0 and 600 as a result. Then, to ensure that the intensity of the gray levels was identical and to prevent negative pixel values from interfering with the calculation of texture features, a set bin width (=1) was used. In order to highlight the difference in ROIs and obtain more high-throughput features, wavelet, gradient, logarithm, exponent, square, square root, and Laplace Gaussian (LoG) filter were used to transform the normalized MR images. The transformation range was 2–6 mm, and the increment was 1 mm. The categories were as follows: 1) first-order features, 2) two-dimensional features, 3) gray-level co-occurrence matrix (GLCM), 4) gray-level dependence matrix (GLDM), 5) gray-level size-zone matrix (GLSZM), 6) gray-level run-length matrix (GLRLM), and 7) neighboring gray tone difference matrix (NGTDM). All the above features were standardized by Z-score to reduce the influence of different dimensions among features. The imaging feature calculation protocol and definition are available online (https://pyradiomics.readthedocs.io/) (25). The ICC of each feature was calculated to avoid the subjective difference in lesion segmentation and ensure repeatability. The features with ICC values ≥0.75 between observers and within observers were selected. Pearson’s correlation coefficients were calculated to identify redundant features. The feature with the largest mean absolute correlation was deleted when the correlation coefficient of the two features was ≥0.9. A least absolute shrinkage and selection operator (LASSO) regression model was used to identify the most representative features, and 10-fold cross-validation was performed (26). Univariate and multivariate logistic regression (LR) analyses were used to choose the clinical independent factors.

### 2.6 Model construction

**Figure 1:**
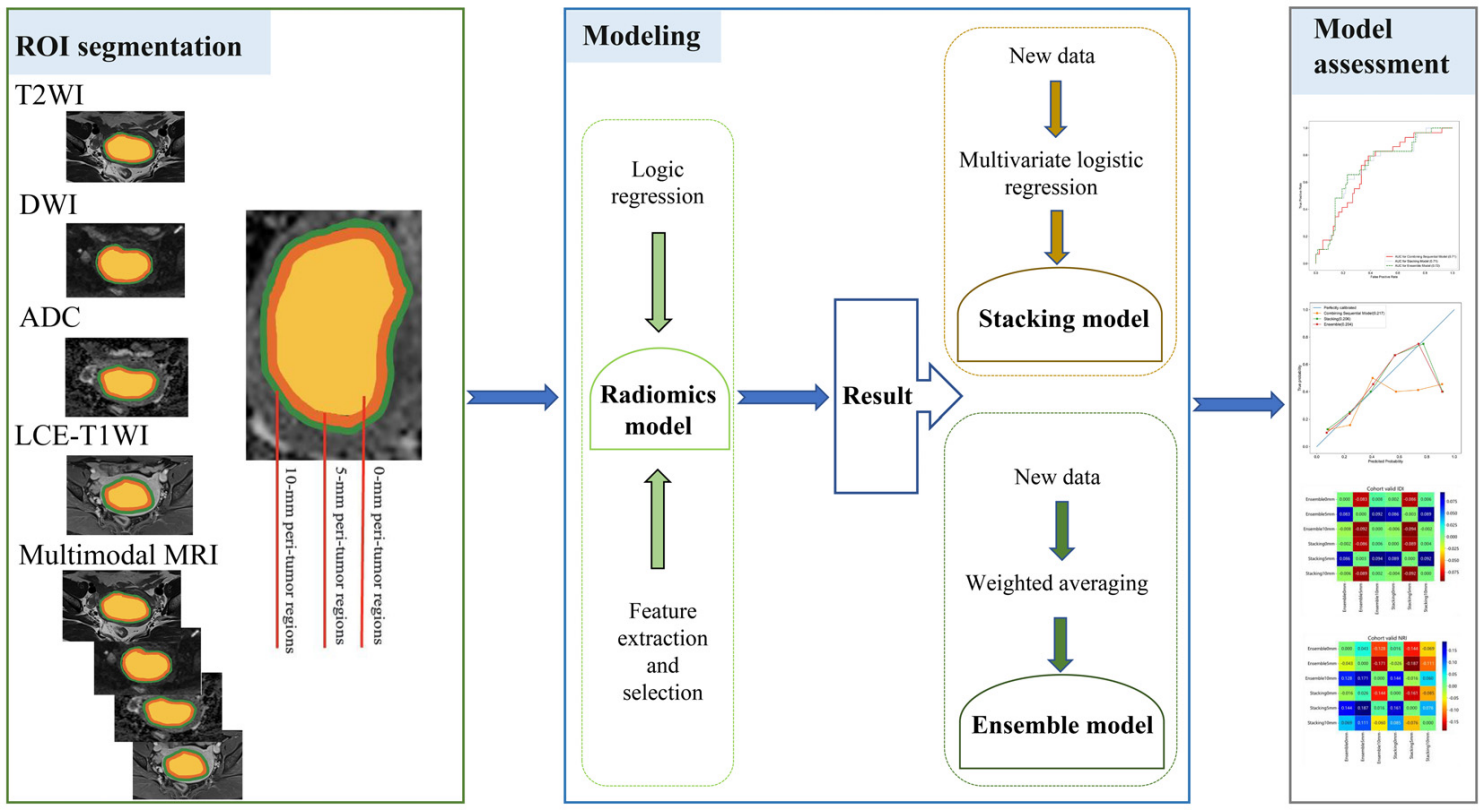
Workflow of this study.

In this study, LR was used to construct models for the features extracted separately from the intratumoral and peritumoral regions expanded by 0 mm, 5 mm, and 10 mm on unimodal and multimodal MRI. A total of 15 models (5 × 3 = 15) were required to be constructed and validated. The stacking model is an ensemble learning technology that integrates many models through a meta-regression model to improve the result prediction accuracy. In this study, a two-tier stacking model was used for the calculation; the first tier used predicted results of the above five models (radiomics models from the ROIs on T2WI, DWI, ADC, LCE-T1WI, and multimodal MRI). The second tier used the results of the first tier as the input of the multivariate LR. Through the meta-regressor, these input features were combined in order to achieve model fusion (27). The ensemble algorithm is developed using the super learner and is an integrated strategy (28). The predicted values were obtained from the above five models using the weighted average method, and the new output was used as the final result. The above model building was implemented in Python (https://www.python.org/getit/), and the detailed process of the model structure adopted is shown in **Figure 1**.

The diagnostic performances of the radiomics models and integrated models were evaluated by sensitivity (SEN), specificity (SPE), accuracy (ACC), and the area under the curve (AUC) of the receiver operating characteristic (ROC) curve. The radiomics model with the highest average AUC for validation groups A and B was considered the optimal radiomics model. DeLong’s tests were performed to evaluate the predictive performance of different models. Clinical decision curve (CDC) was used to estimate the clinical net benefit of the radiologist and different models.

### 2.7 Statistical analyses

Statistical analysis was conducted using SPSS 26.0 (IBM, Armonk, NY, USA), R software 4.1.2 (https://www.r-project.org/), and Python 3.9.7 (https://www.python.org/). Categorical variables and continuous variables were respectively expressed as means value ± standard deviation and counts. Categorical variables were analyzed using the chi-square test or Fisher’s exact test, whereas continuous variables were evaluated using one-way ANOVA, the Mann–Whitney U test, or the Kruskal–Wallis test. Univariate and multivariate LR analyses were used to filter the clinical predictors. Statistical significance was set at *p* < 0.05. Pearson’s correlation analyses were used to evaluate correlations between continuous variables. It’s considered to be correlations between the variables if *p* < 0.05.

## 3 Results

### 3.1 Clinical parameters

The clinical information of the patients in the training group and the validation groups is summarized in **Table 2**. Age, menopausal status, and CA199 were found to be reliable predictors of DMI in early-stage EAC by univariate LR analysis. Multivariate LR analysis showed that menopausal status and CA199 remained independent predictors of DMI in early-stage EAC (*p* < 0.05).

**Table 2:** Clinical characteristics of patients in the training group and validation group.

	Training group (N = 281)		Validation group A (N = 71)		Validation group B (N = 107)	
	IA	IB	IA	IB	IA	IB
Number	222	59	41	30	78	29
Age (mean ± SD)	52.03 ± 8.73	56.86 ± 8.44	54.80 ± 9.58	53.17 ± 8.65	51.78 ± 8.23	56.37 ± 5.68
CA125	30.99 ± 60.67	63.59 ± 98.82	30.20 ± 46.92	63.42 ± 230.96	16.02 ± 13.66	26.43 ± 21.67
CA199	30.18 ± 71.81	142.80 ± 325.64	30.64 ± 29.83	40.99 ± 50.07	21.41 ± 11.20	27.59 ± 19.82
BMI (kg/m^2^)	24.77 ± 4.23	24.23 ± 3.57	24.64 ± 3.84	24.67 ± 3.29	25.46 ± 4.37	25.53 ± 4.54
Menopause						
Yes	125 (56.31%)	50 (84.75%)	23 (56.10%)	19 (63.33%)	37 (47.44%)	22 (75.86%)
No	97 (43.69%)	9 (15.25%)	18 (43.90%)	11 (36.67%)	41 (52.56%)	7 (24.14%)
Metabolic syndrome						
Yes	69 (31.08)	24 (40.68%)	10 (24.39%)	4 (13.33%)	33 (42.31%)	12 (41.38%)
No	153 (68.92%)	35 (59.32%)	31 (75.61%)	26 (86.67%)	45 (57.69%)	17 (58.62%)

FIGO, International Federation of Gynecology and Obstetrics; SD, standard deviation; BMI, body mass index; CA125, cancer antigen 125; CA199, cancer antigen 199.

### 3.2 Feature selection and performance of radiomics models

Among all the extracted features, 608 features (intratumoral regions: henceforth, 0 mm), 479 features (intratumoral and peritumoral regions expanded by 5 mm: henceforth, 5 mm), and 709 features (intratumoral and peritumoral regions expanded by 10 mm: henceforth, 10 mm) were excluded based on ICC values less than 0.75 either between or within observers. After Pearson’s correlation analysis, 5,267 features (0 mm), 5,344 features (5 mm), and 4,992 features (10 mm) were excluded. After the LASSO classifier, the top four features from the T2WI, DWI, ADC, LCE-T1WI, and multimodal MRI were screened out. The selected features and weights are shown in **Supplementary Material 1**-**4**. The RadScores were calculated based on the coefficients and intercepts obtained from the LR models.

**Figure 2:**
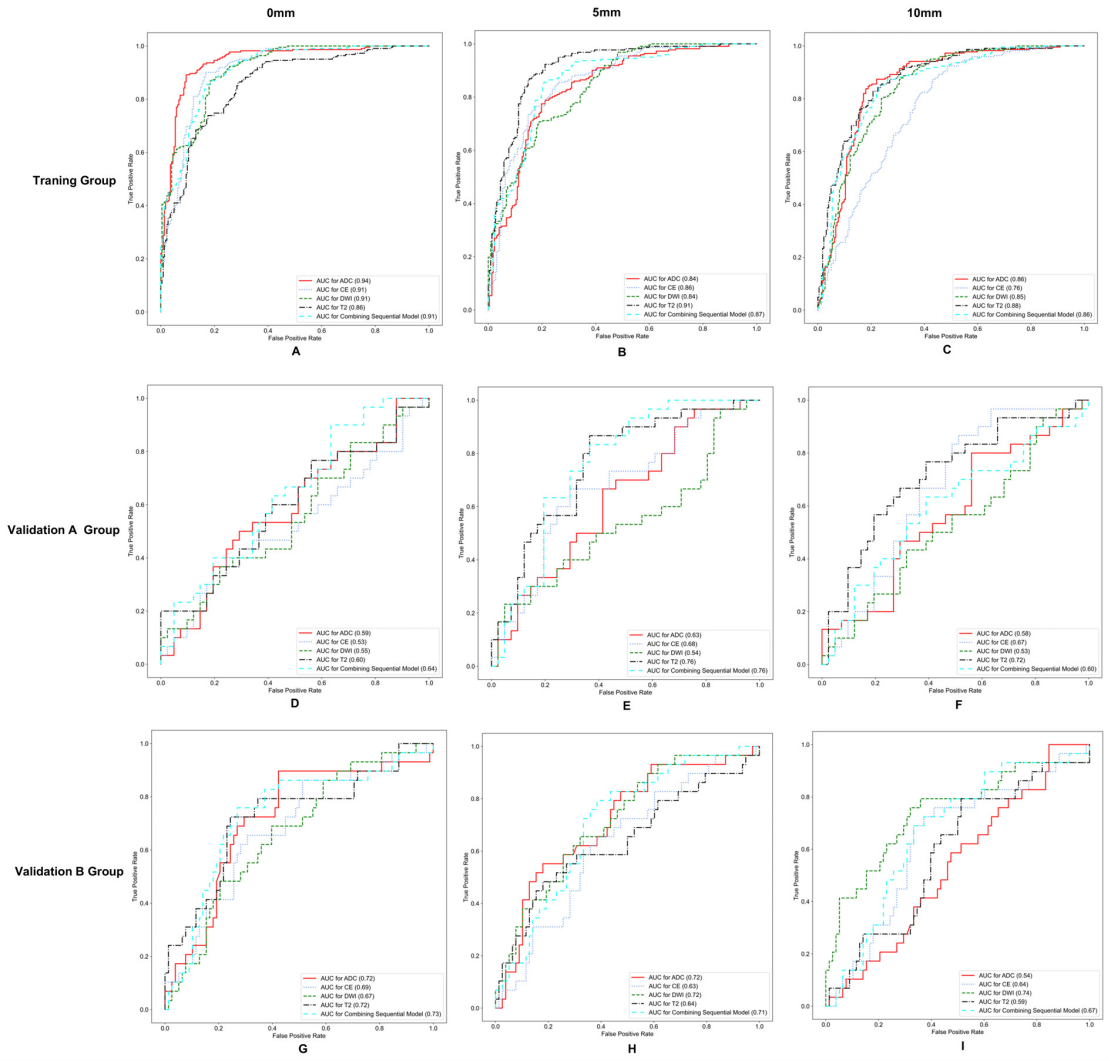
Receiver operating characteristic (ROC) curves for different models in the training group **(A–C)**, validation group A **(D–F)**, and validation group B **(G–I)**.

The AUC, ACC, SEN, and SPE of radiomics models are shown in **Table 3**, and the ROC curves in the training group, validation group A, and validation group B are presented in **Figure 2**. The optimal radiomics model was the multimodal MRI-based radiomics model from the intratumoral and peritumoral regions expanded by 5 mm, with an AUC of 0.74 in the validation group. In the optimal radiomics model, the features that contribute most were as follows:

**Table 3:** Prediction performance of various radiomics models for the determination of deep myometrial invasion.

Sequence		Training group N = 281			Validation group A N = 71			Validation group B N = 107			Validation group N = 178		
		0 mm	5 mm	10 mm	0 mm	5 mm	10 mm	0 mm	5 mm	10 mm	0 mm	5 mm	10 mm
T2WI	AUC	0.86	0.91	0.88	0.60	0.76	0.72	0.73	0.64	0.59	0.67	0.70	0.67
	ACC	0.76	0.86	0.80	0.56	0.65	0.69	0.73	0.72	0.58	0.65	0.69	0.64
	SEN	0.75	0.88	0.81	0.37	0.60	0.53	0.62	0.48	0.41	0.50	0.54	0.47
	SPE	0.77	0.83	0.79	0.71	0.68	0.80	0.77	0.81	0.64	0.74	0.75	0.72
LCE-T1WI	AUC	0.91	0.86	0.76	0.53	0.68	0.67	0.69	0.63	0.64	0.61	0.66	0.66
	ACC	0.86	0.79	0.68	0.58	0.68	0.59	0.68	0.63	0.66	0.63	0.66	0.63
	SEN	0.89	0.79	0.70	0.47	0.57	0.47	0.55	0.52	0.66	0.51	0.55	0.57
	SPE	0.83	0.78	0.67	0.66	0.76	0.68	0.73	0.67	0.67	0.70	0.72	0.68
DWI	AUC	0.91	0.84	0.85	0.55	0.54	0.53	0.67	0.72	0.74	0.61	0.63	0.63
	ACC	0.84	0.73	0.77	0.54	0.56	0.55	0.65	0.71	0.71	0.60	0.64	0.63
	SEN	0.88	0.76	0.81	0.40	0.40	0.33	0.55	0.59	0.66	0.48	0.50	0.50
	SPE	0.80	0.69	0.73	0.63	0.68	0.71	0.69	0.74	0.72	0.66	0.71	0.72
ADC	AUC	0.94	0.84	0.86	0.59	0.63	0.58	0.72	0.72	0.54	0.66	0.68	0.56
	ACC	0.89	0.78	0.82	0.62	0.61	0.58	0.68	0.67	0.59	0.65	0.64	0.59
	SEN	0.91	0.79	0.85	0.37	0.50	0.40	0.34	0.62	0.28	0.34	0.56	0.34
	SPE	0.86	0.77	0.80	0.80	0.68	0.71	0.81	0.69	0.71	0.81	0.69	0.71
Multimodality	AUC	0.91	0.87	0.86	0.64	0.76	0.60	0.73	0.71	0.67	0.69	0.74	0.64
	ACC	0.84	0.82	0.79	0.59	0.65	0.61	0.75	0.67	0.65	0.67	0.66	0.63
	SEN	0.86	0.85	0.79	0.30	0.43	0.40	0.62	0.55	0.72	0.46	0.49	0.56
	SPE	0.81	0.79	0.79	0.81	0.80	0.76	0.79	0.72	0.63	0.80	0.76	0.70

T2WI, T2-weighted imaging; LCE-T1WI, late contrast-enhanced T1-weighted imaging; DWI, diffusion-weighted imaging; ADC, the apparent diffusion coefficient; AUC, area under the curve; ACC, accuracy; SEN, sensitivity; SPE, specificity.

T2_log_sigma_6_0_mm_3D_gldm_Dependence_NonUniformityNormalized,

CE_gradient_gldm_SmallDependence_LowGrayLevel_Emphasis,

CE_log_sigma_3_0_mm_3D_gldm_LargeDependence_HighGrayLevel_Emphasis,

and ADC_log_sigma_6_0_mm_3D_firstorder_Maximum.

### 3.3 Performance and clinical application of integrated model

**Figure 3:**
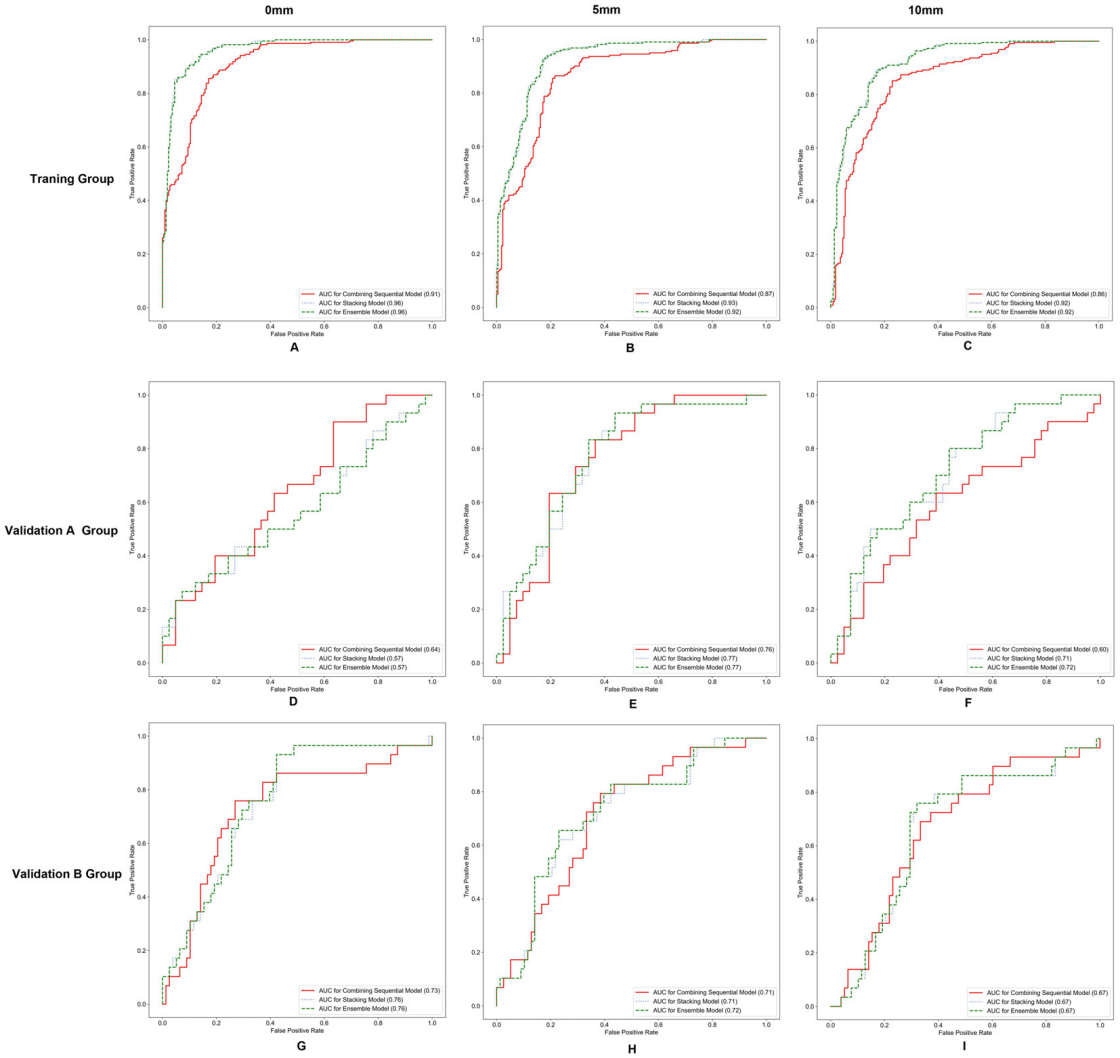
Receiver operating characteristic (ROC) curves for different integrated models in the training group **(A-C)**, validation group A **(D-F)**, and validation group B **(G-I)**.

**Figure 4:**
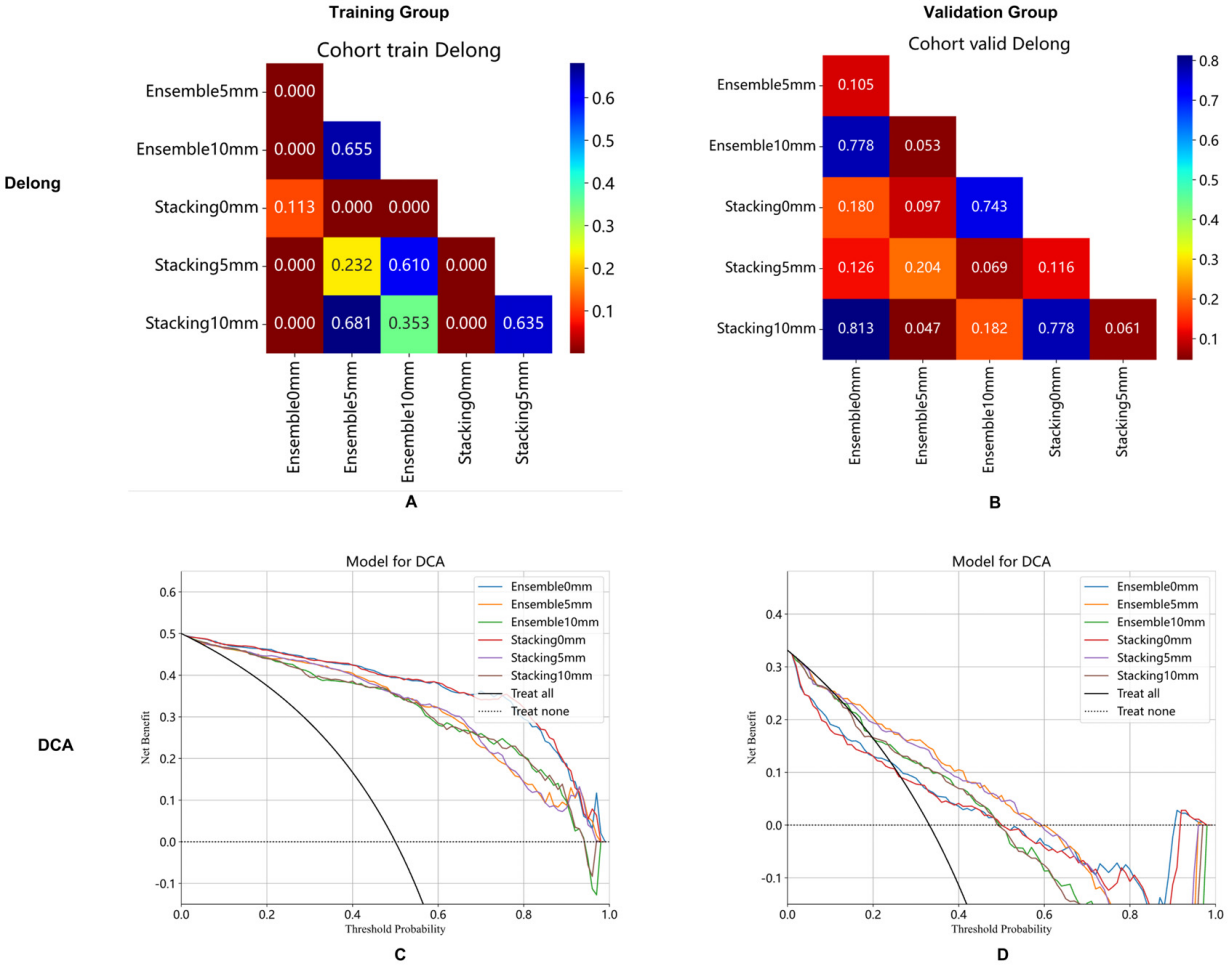
Ensemble model and stacking model of DeLong's test **(A, B)** and clinical decision curve **(C, D)** with 0 mm, 5 mm, and 10 mm of peritumoral regions in the training group **(A, C)** and validation cohort **(B, D)**.

In the training group, the AUCs of the stacking model were 0.96, 0.93, and 0.92 for 0 mm, 5 mm, and 10 mm, respectively. The AUCs of the ensemble model were 0.96, 0.92, and 0.92 for 0 mm, 5 mm, and 10 mm, respectively. In the validation group, the AUCs of the stacking model were 0.67, 0.74, and 0.69 for 0 mm, 5 mm, and 10 mm, respectively. The AUCs of the ensemble model were 0.67, 0.75, and 0.70 for 0 mm, 5 mm, and 10 mm, respectively. When the same integrated algorithm was used, the integrated models with 5-mm expansion presented higher AUCs than those with 0-mm and 10-mm expansion in the validation group. The performance of the stacking model and ensemble model with 5-mm expansion was similar, and their AUCs were 0.74 and 0.75, respectively. The performance of each model is presented in **Table 4**. ROC curves in the training group, validation group A, and validation group B are presented in **Figure 3**. The heat maps of DeLong’s test and CDCs of the ensemble model and stacking model are shown in **Figure 4**. The heat map of DeLong’s test showed that there was little difference between the integrated models in the validation group. According to CDCs, the ensemble model with 5-mm expansion for predicting the DMI of early-stage EAC showed clinical net benefit in the validation group.

**Table 4:** Prediction performance of different integrated radiomics models.

Group	Models	AUC (95%CI)	ACC (%)	SEN (%)	SPE (%)
Training group	Stacking mode (0 mm)	0.96	0.90	0.92	0.87
	Stacking model (5 mm)	0.93	0.86	0.87	0.84
	Stacking model (10 mm)	0.92	0.85	0.87	0.84
	Ensemble model (0 mm)	0.96	0.89	0.92	0.87
	Ensemble model (5 mm)	0.92	0.85	0.87	0.84
	Ensemble model (10 mm)	0.92	0.85	0.86	0.83
Validation group A	Stacking model (0 mm)	0.57	0.62	0.33	0.83
	Stacking model (5 mm)	0.77	0.66	0.53	0.76
	Stacking model (10 mm)	0.71	0.68	0.40	0.88
	Ensemble model (0 mm)	0.57	0.62	0.34	0.83
	Ensemble model (5 mm)	0.77	0.69	0.57	0.78
	Ensemble model (10 mm)	0.72	0.68	0.43	0.85
Validation group B	Stacking model (0 mm)	0.76	0.71	0.38	0.82
	Stacking model (5 mm)	0.71	0.75	0.48	0.85
	Stacking model (10 mm)	0.67	0.65	0.52	0.71
	Ensemble model (0 mm)	0.76	0.70	0.38	0.82
	Ensemble model (5 mm)	0.72	0.75	0.48	0.85
	Ensemble model (10 mm)	0.67	0.65	0.52	0.71
Validation group	Stacking model (0 mm)	0.67	0.67	0.92	0.87
	Stacking model (5 mm)	0.74	0.71	0.87	0.84
	Stacking model (10 mm)	0.69	0.67	0.87	0.84
	Ensemble model (0 mm)	0.67	0.66	0.92	0.87
	Ensemble model (5 mm)	0.75	0.72	0.87	0.84
	Ensemble model (10 mm)	0.70	0.67	0.86	0.83

AUC, area under the curve; ACC, accuracy; SEN, sensitivity; SPE, specificity.

## 4 Discussion

In this study, we found that the optimal radiomics model was the multimodal MRI-based radiomics model from the intratumoral and peritumoral regions expanded by 5 mm. However, the predictive performance of the integrated models did not improve compared with that of the simple radiomics models, and the ensemble model with 5-mm expansion for predicting the DMI of early-stage EAC demonstrated clinical net benefit in the validation group.

Some studies have lately started to investigate peritumoral areas in the hopes of providing more additional and helpful information about the tumors, given the expanding understanding of the biological behavior and the underlying microenvironment of tumors (29). The peritumoral region was found to be highly linked with tumor invasion in earlier radiomics studies (30, 31).

Therefore, we constructed intratumoral and peritumoral radiomics models to predict the DMI of early-stage EA. Lei et al. (32) found that ADC values in the 5-mm peritumoral region may be helpful in differentiating between DMI and superficial myometrial infiltration (22). Niha Beig et al. showed that radiomics characteristics located approximately 5 mm outside the tumor can be extracted to differentiate adenocarcinoma from granuloma (33). Tumor-associated macrophages and lymphocytes infiltrating the tumor were discovered to be closely packed “edges” of the tumor interface in the representative hematoxylin and eosin staining images (33). Our study showed that the multimodal radiomics model with 5-mm expansion acquired the highest AUC. The AUCs of the radiomics models with 10-mm expansion were not high, possibly because the overlarge peritumoral regions contained normal tissue, which caused the model’s performance to collapse (34). Wu et al. (34) indicated that stable performance of the radiomics model was achieved only in 1.5- to 4.5-mm tumors in the peritumoral regions.

In this study, compared to the unimodal radiomics models, the multimodal radiomics models with 0 mm and 5-mm expansion obtained higher AUCs in the validation group. Numerous studies have shown that the performance of the multimodal MRI-based radiomics models was superior to that of the unimodal radiomics models (35, 36). Wang et al. (37) considered that multimodal radiomics models performed better in differentiating between subtypes of early cervical cancer than any unimodal radiomics models. Combining multiparametric MRI features could enhance prediction because distinct sequences represented the various biological characteristics of the tumor, such as tumor composition, cellularity, and vascularization (38).

A recent study indicated that the stacking model demonstrated strong stability and achieved great diagnostic performance (39, 40). Nevertheless, the performance of the integrated models was not improved compared with that of the simple radiomics models in this study. The benefit of the ensemble approach is that it can enhance the model’s generalization and resilience in classification and prediction while lowering the model’s variance and bias through a strong majority voting or group average procedure (41). We found that the ensemble model with 5-mm expansion had a clinical net benefit in the validation group, which suggested that the integrated approach may have the potential to improve clinical effectiveness and needed to be validated with larger sample sizes in the future.

This study has some limitations. First, although manual segmentation is the gold standard, it is time-consuming. In the future, we will develop deep learning algorithms that can segment tumors automatically. Second, this was a retrospective study, which may cause potential selection bias. In the future, prospective validation will be performed. Third, although N4 bias field correction has been performed, there may be potential impacts on model results due to differences in scanners and parameters in multi-center studies.

## 5 Conclusion

The MRI-based radiomics models that radiomics features extracted from both intra- and peritumoral regions have the potential to improve the performance for detecting DMI in early-stage EAC patients before surgery, and the multimodal radiomics model with 5-mm expansion has the best performance. The integrated models with stacking and ensemble algorithms have little value in improving the prediction.

## Data Availability

The original contributions presented in the study are included in the article/**Supplementary Material**. Further inquiries can be directed to the corresponding author.
