# Supplementary material for: MRI-based intratumoral and peritumoral radiomics for assessing deep myometrial invasion in patients with early-stage endometrioid adenocarcinoma
Source: Front Oncol. 2025 Jan 15;14:1474427. doi: 10.3389/fonc.2024.1474427 (PMC11774896; doi:10.3389/fonc.2024.1474427)
Supplement: Supplementary file 1 [file DataSheet1.docx]

| 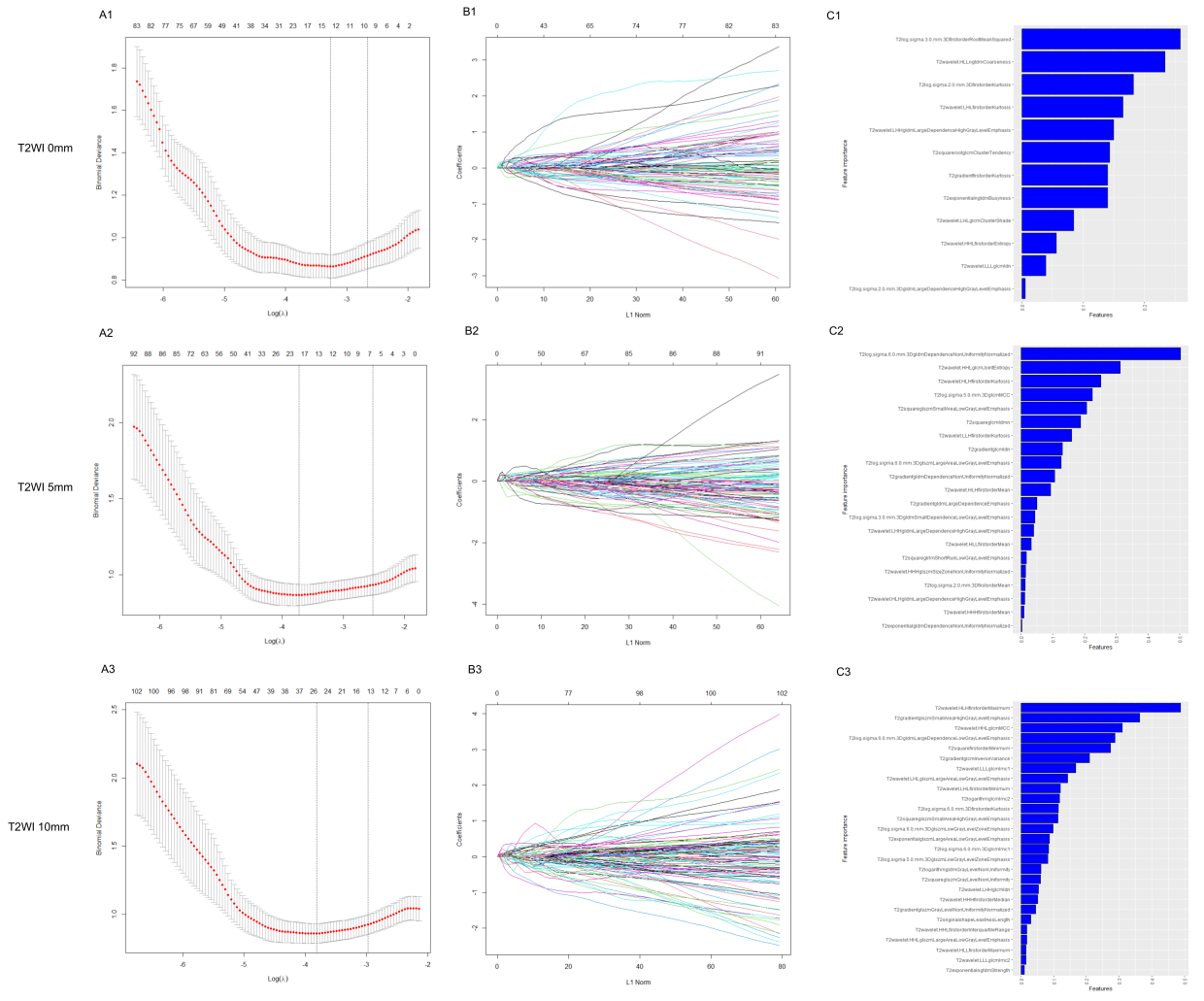 |
| --- |
| Supplemental material 1  Feature selection using the least absolute shrinkage and selection operator (LASSO) regression model. The cross-validation plot (A), the coefficient profile plot (B) and bar chart of feature weight (C) on T2-weighted imaging (T2WI) with peritumoral expansion at different distances. |

| 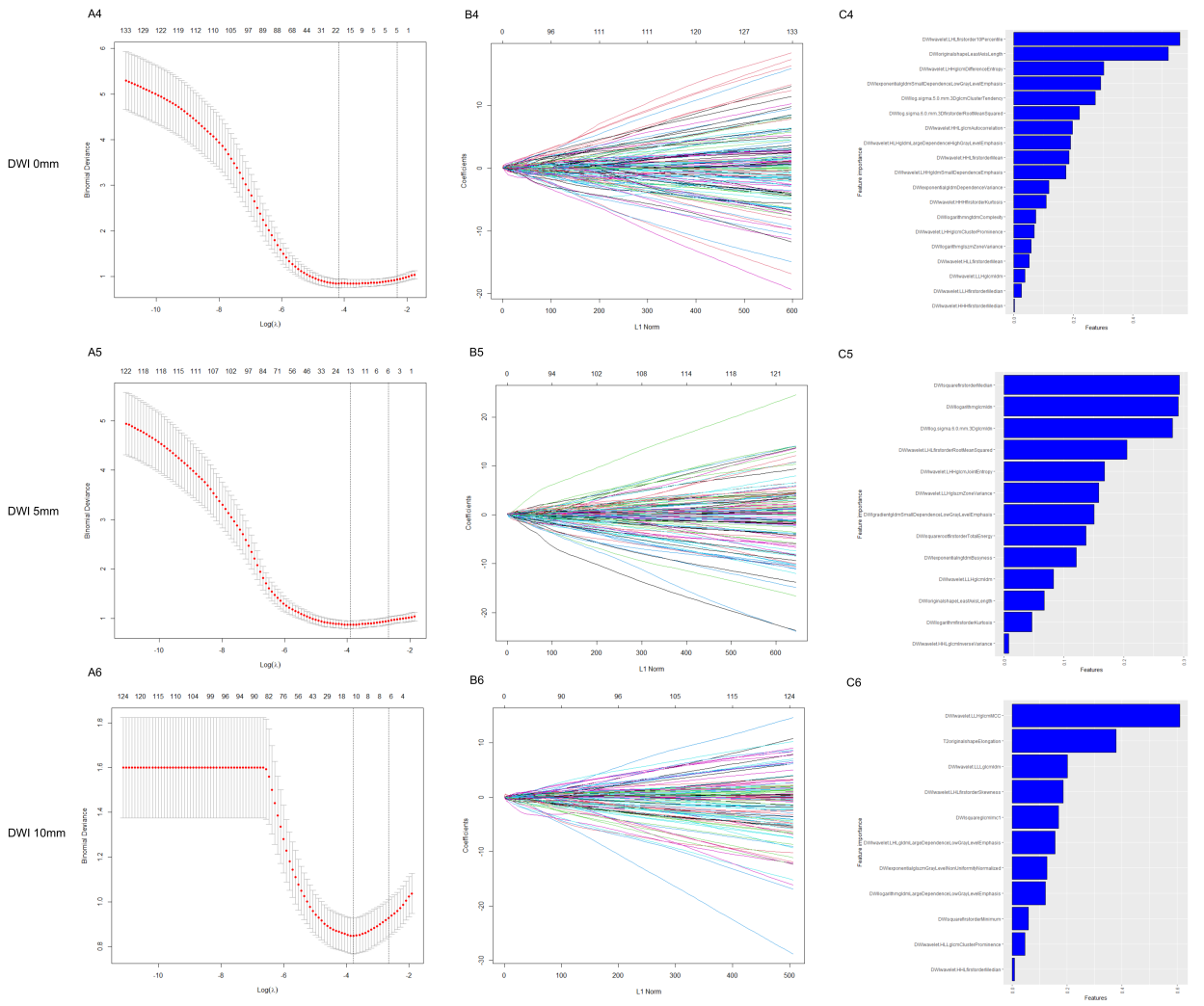 |
| --- |
| Supplemental material 2  Feature selection using the least absolute shrinkage and selection operator (LASSO) regression model. The cross-validation plot (A), the coefficient profile plot (B) and bar chart of feature weight (C) on diffusion-weighted imaging (DWI) with peritumoral expansion at different distances. |

| 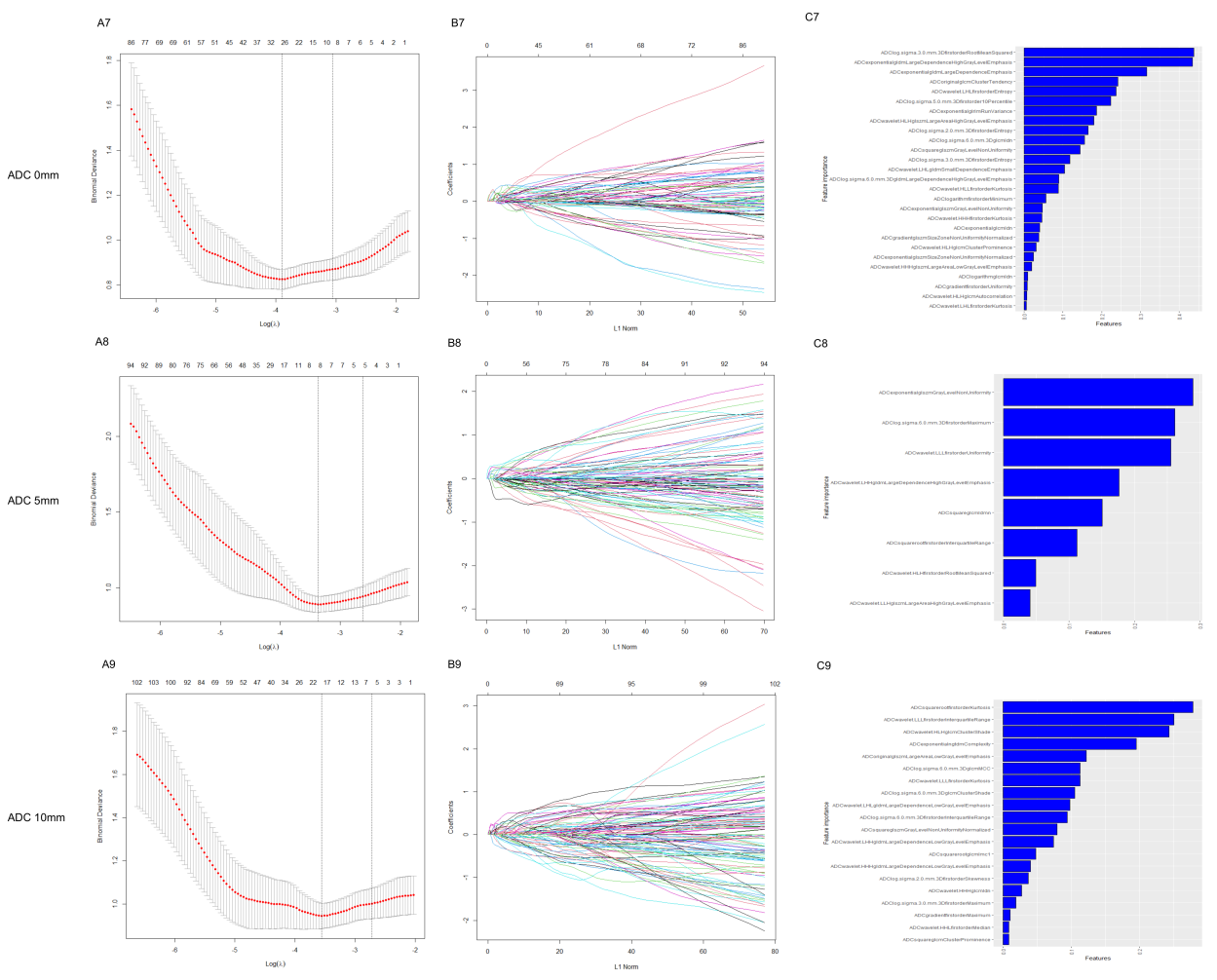 |
| --- |
| Supplemental material 3  Feature selection using the least absolute shrinkage and selection operator (LASSO) regression model. The cross-validation plot (A), the coefficient profile plot (B) and bar chart of feature weight (C) on apparent diffusion coefficient (ADC) maps with peritumoral expansion at different distances. |

| 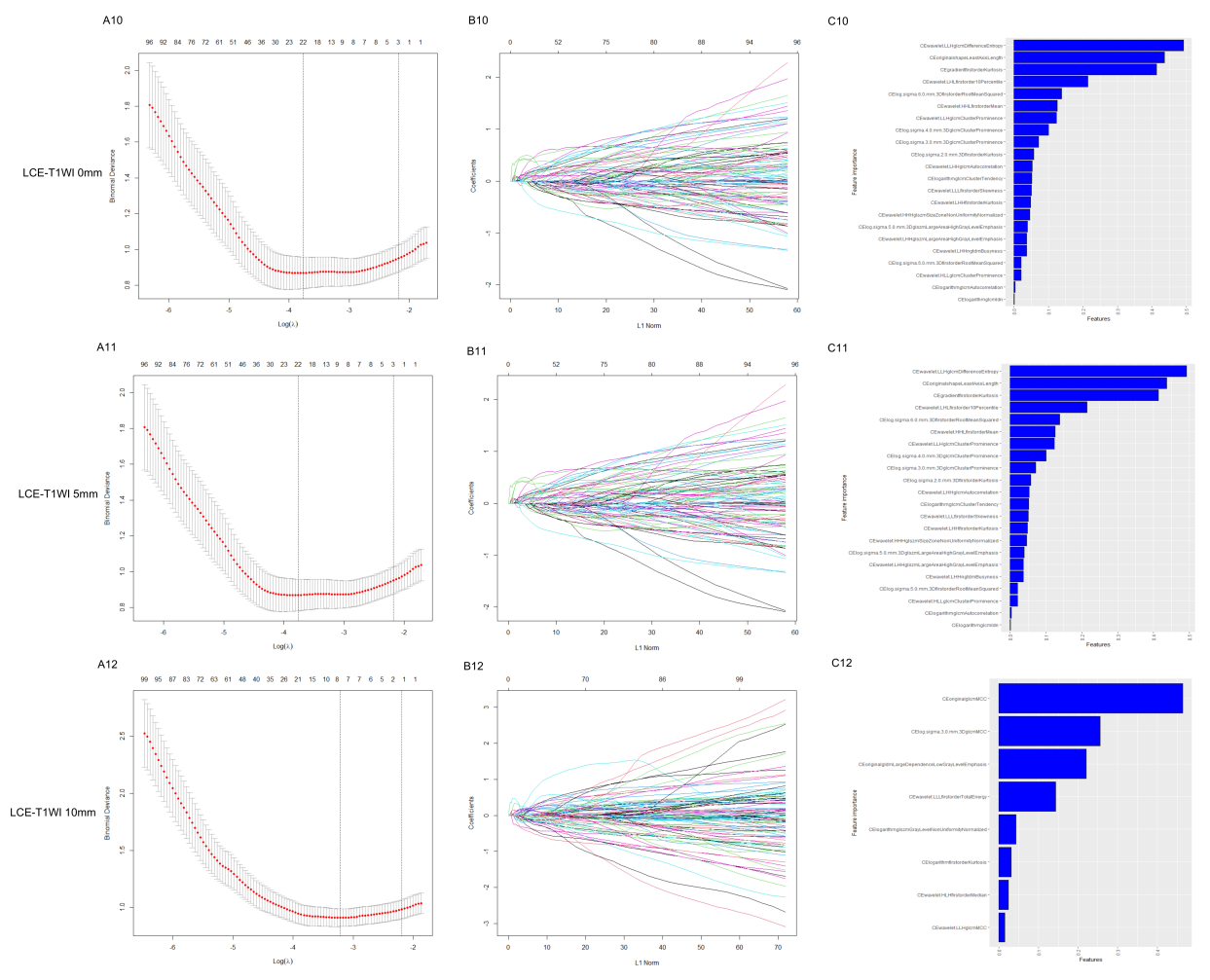 |
| --- |
| Supplemental material 4  Feature selection using the least absolute shrinkage and selection operator (LASSO) regression model. The cross-validation plot (A), the coefficient profile plot (B) and bar chart of feature weight (C) on late contrast-enhanced T1-weighted imaging (LCE-T1WI) with peritumoral expansion at different distances. |

| 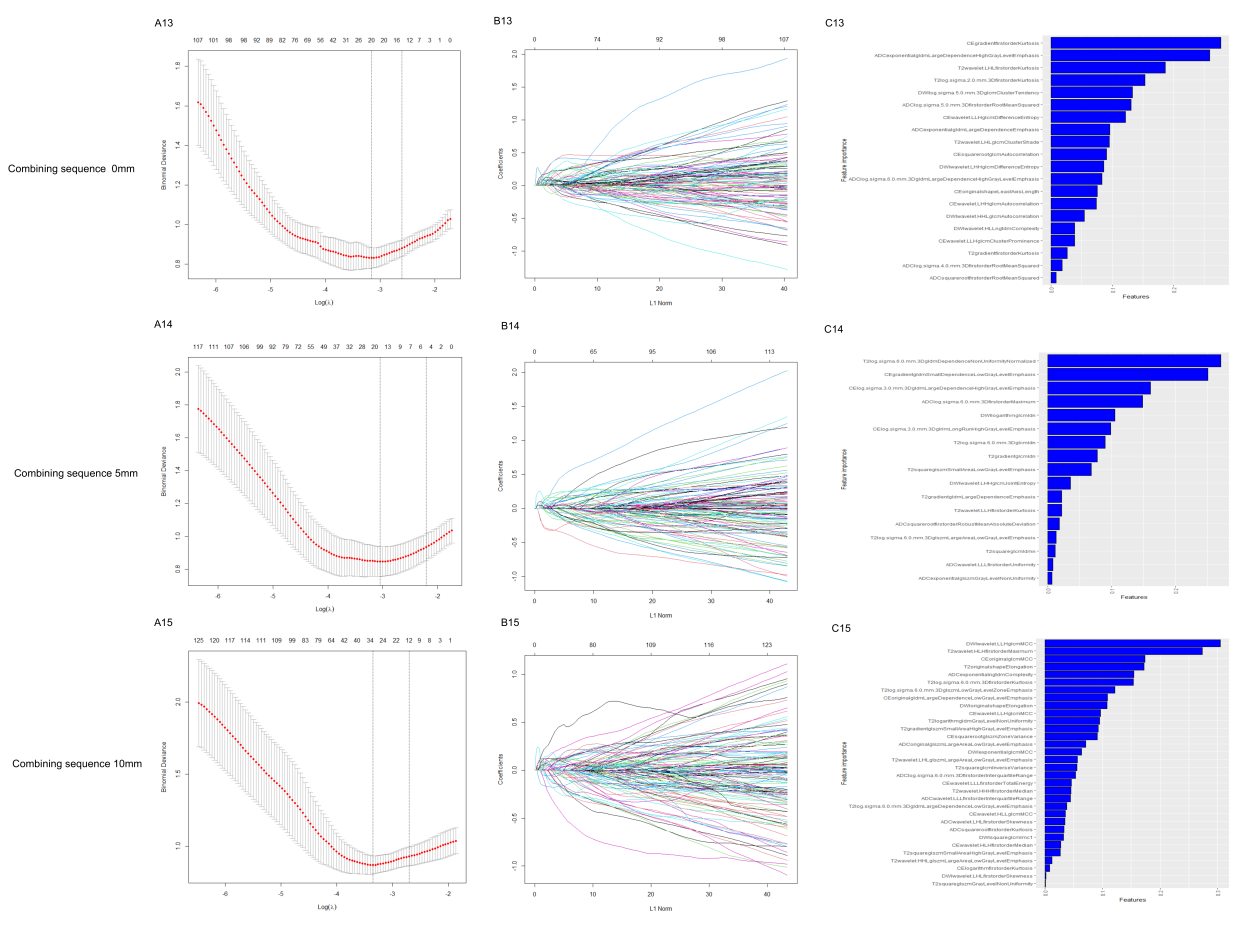 |
| --- |
| Supplemental material 5  Feature selection using the least absolute shrinkage and selection operator (LASSO) regression model. The cross-validation plot (A), the coefficient profile plot (B) and bar chart of feature weight (C) on multimodal MRI with peritumoral expansion at different distances. |
